# Pharmaceutical procurement among public sector procurers in CARICOM

**DOI:** 10.26633/RPSP.2021.57

**Published:** 2021-05-20

**Authors:** Charles Preston, Claire King, Maryam Hinds, Francis Burnett, Rian Marie Extavour

**Affiliations:** 1 Ex officer of Pan American Health Organization Washington, D.C United States of America Ex officer of Pan American Health Organization, Washington, D.C., United States of America; 2 Baylor Scott and White Medical Center Taylor, Tex United States of America Baylor Scott and White Medical Center, Taylor, Tex., United States of America; 3 Barbados Drug Service St. Michael Barbados Barbados Drug Service, St. Michael, Barbados; 4 Organisation of Eastern Caribbean States Castries Saint Lucia Organisation of Eastern Caribbean States, Castries, Saint Lucia; 5 Caribbean Public Health Agency Port of Spain Trinidad and Tobago Caribbean Public Health Agency, Port of Spain, Trinidad and Tobago

**Keywords:** Access to essential medicines and health technologies, pharmaceutical trade, drug industry, economics, pharmaceutical, Caribbean region, Acceso a medicamentos esenciales y tecnologías sanitarias, comercialización de medicamentos, industria farmacéutica, economía farmacéutica, region del Caribe, Acesso a medicamentos essenciais e tecnologias em saúde, comercialização de medicamentos, indústria farmacêutica, farmacoeconomia, região do Caribe

## Abstract

**Objective.:**

To examine multiple aspects of the medicines in CARICOM procurement markets, including manufacturer headquarters location, regulatory history, and type (innovator versus generic); the proportion of World Health Organization (WHO) essential medicines; and the most expensive medicines procured.

**Methods.:**

An analysis of procurement information from selected CARICOM procurers. Four public sector procurement lists were obtained based on public availability or sharing of data from public sector procurers. Analyses were based on parameters available or deduced from these data.

**Results.:**

The majority of products come from manufacturers headquartered in North America and Europe (63%–67%). The percentage of medicines procured from generic companies is 60%–87%; and 25%–50% of medicines procured are on the WHO Essential Medicines List. Wide price variations exist in the most expensive medicines purchased.

**Conclusions.:**

The analysis identifies vulnerabilities and opportunities in the procurement situation of CARICOM states, particularly related to quality and rational use of medicines. This analysis represents a baseline that governments and other stakeholders can use in the future.

## RESUMEN

**Objetivo.:**

Revisar los múltiples aspectos de los medicamentos en los mercados de compras y los proveedores de CARICOM, como la ubicación de la sede del fabricante, el historial de regulación, el tipo (patentado versus genérico); la proporción de medicamentos esenciales de la Organización Mundial de la Salud (OMS); y los medicamentos comprados más caros.

**Métodos.:**

Se analizó información sobre la compra por parte de determinados organismos de CARICOM. La información procedía de cuatro listas de organismos del sector público que realizan las compras, que se consiguieron en función de su disponibilidad pública o de los datos distribuidos por los organismos del sector público que realizan las compras. Los análisis estaban basados en los parámetros disponibles o derivados de estos datos.

**Resultados.:**

La mayoría de los productos proviene de fabricantes radicados en América del Norte y Europa (entre 63% y 67%). El porcentaje de medicamentos que se compra de empresas genéricas oscila entre 60% y 87%; y de 25% a 50% de los medicamentos que se compran están en la Lista de Medicamentos Esenciales de la OMS. Hay una gran divergencia de precios entre los medicamentos comprados más caros.

**Conclusiones.:**

En el análisis se han encontrado vulnerabilidades y oportunidades con respecto a la situación de las compras de medicamentos de los Estados de CARICOM, especialmente en cuanto a la calidad y al uso racional de los medicamentos. Este análisis representa una línea de base que los gobiernos u otros interesados directos pueden utilizar en el futuro.

## RESUMO

**Objetivo.:**

Examinar vários aspectos relacionados aos mercados e fornecedores de produtos farmacêuticos da CARICOM, incluindo a localização da sede do laboratório fabricante, histórico regulatório e tipo de produtos (inovadores versus genéricos); proporção de medicamentos adquiridos que constam da relação de medicamentos essenciais da Organização Mundial da Saúde (OMS); e medicamentos mais caros comprados.

**Métodos.:**

Foi realizada uma análise de informação sobre compras feitas por compradores selecionados da CARICOM. Quatro listas de compras do setor público foram obtidas com informação de acesso público ou compartilhada pelos compradores. As análises foram feitas com base em parâmetros disponíveis ou inferidos a partir dos dados.

**Resultados.:**

A maioria dos produtos farmacêuticos é proveniente de laboratórios com sedes na América do Norte e Europa (63%–67%). Do total, 60%–87% dos medicamentos adquiridos são de laboratórios de produtos genéricos e 25%–50% constam da relação de medicamentos essenciais da OMS. Existe uma ampla variação nos preços dos medicamentos mais caros comprados.

**Conclusões.:**

Foram identificadas fragilidades e oportunidades na situação de compras dos países da CARICOM, em particular relacionadas à qualidade dos produtos e ao uso racional dos medicamentos. Esta análise serve de referência a ser usada futuramente pelos governos e outras partes interessadas.

Access to medicines is a fundamental tenet of universal health and key to achievement of the Sustainable Development Goals of the United Nations. However, there is a scarcity of published analyses on this topic in the peer-reviewed literature from the Caribbean Community^1^ (CARICOM), which consists of 20 English-speaking governments, plus Haiti and Suriname. The timing is important because of intense focus on medicines issues at the regional and global levels. A 2019 report by the Director General of the World Health Organization (WHO) noted that there have been more than 100 resolutions of the World Health Assembly and the Regional Committee related to the subject in the last 10 years (1), including a Pan American Health Organization Resolution on Access and Rational Use of Strategic and High-cost Medicines and Other Health Technologies (2). Because of their small size, Caribbean nations face special challenges related to medicines, including smaller and less attractive markets, and smaller economies that limit available financial resources.

There have also been significant pharmaceutical policy developments in CARICOM over the last decade. These include the establishment of a Caribbean Pharmaceutical Policy (3), the creation of a pharmaceutical policy advisory committee (TECHPHARM), and the establishment of a subregional mechanism for medicines regulation called the Caribbean Regulatory System (CRS), managed by the Caribbean Public Health Agency (CARPHA) (4). These actions point to a growing need to better understand the pharmaceutical situation as a benchmark for future progress.

Broader knowledge of the pharmaceutical situation in CARICOM comes from various data sources, including pharmaceutical country profiles (5) and regulatory system analyses (6). Yet there is a lack of synthesized information about medicines that are on the markets of the various Member States. Information on medicines authorized for legal sale is difficult to analyze as few CARICOM countries publish their registries (7). However, all 14 sovereign governments procure medicines for the public sector through national or regional procurement agencies (8), and some governments publish lists of procured medicines.

The data in these lists may provide important information. Although there is variation in what countries publish, many of the parameters are common across countries, which enables comparisons. The data are also published on a regular basis, making it a recurring source of information. More specifically, these lists typically include information on molecules, doses (strength), formulations, manufacturers, and prices. With this information, it is possible to make deductions about additional parameters. For example, it can provide insight into the potential quality of products. It can also help to understand rational use issues, such as the extent they are listed on the WHO Essential Medicines List (WHO EML) (9).

This study examines multiple aspects of CARICOM procurement markets and suppliers, including the location of manufacturer headquarters; the regulatory history of manufacturers; the types of manufacturers (innovator versus generic); the proportion of WHO essential medicines; and the most expensive medicines procured.

## MATERIALS AND METHODS

Public sector procurement lists (10–12) were selected for analysis based on public availability or sharing of data from national/regional procurers, and have been anonymized as procurers A, B, C, and D. The lists represent public authorities that serve about 5 million out of the 7 million people in the English-speaking part of CARICOM.

### Identification and consolidation of procurement data

Procurement lists were identified using Internet searches for the name of each CARICOM state and search terms including “drugs,” “medicines,” “procurement list,” “prices,” and “tenders.” Products that were not defined as medicines according to the WHO’s definition were excluded from the review, namely: vaccines, diagnostic agents, disinfectants, and antiseptics.

### Development of a procurement database

Information on molecule (active ingredient), dose, formulation, manufacturer, and price was extracted and consolidated into an Excel database. Supplemental procurement information on the location of the manufacturer headquarters, regulatory history, company type, WHO essential medicines, and price was added and analyzed according to the following categories:

**Location of manufacturer headquarters.** This parameter was based on the address of the headquarters of the manufacturer as identified on the manufacturer’s website. If not found, the location of the parent company was used. If no parent company was found, the authors used the company’s location listed on the original procurement list. Note, the location of the plant that makes a particular product has more bearing on quality; however, these data are typically included only in confidential regulatory findings, and headquarters gives a rough indication of the geography of manufacturers supplying the Caribbean.

**Regulatory history.** Regulatory history of the manufacturer describes whether the manufacturer had at least one prescription medicine approved by a reference regulatory authority (RRA). Note the terms “approval,” “registration,” and “market authorization” are used interchangeably (13). Each manufacturer’s name was searched in the online product marketing authorization/registration/approval databases of the regulatory authorities of Australia, Canada, the United Kingdom, and the United States of America. If the specified manufacturer was not found, the authors searched for a parent company. The listed regulatory authorities were selected because of their history as Stringent Regulatory Authorities (SRAs) (14), their common historical and linguistic ties to the Caribbean, and their citations in a number of national pharmaceutical laws in the Caribbean. WHO’s Prequalification of Medicines program could also be used to check regulatory history, as it represents an important international quality standard, and Caribbean countries have begun to recognize this program.

**Company type.** Company type was determined using available information on the manufacturer’s website, where the type of medicine produced was indicated (e.g., generic versus originator). In the case of a mixed portfolio, the authors defaulted to the type of product that constitutes the majority of the manufacturer’s product line.

**WHO EML.** A medicine was considered essential if it appeared on the WHO’s Model List of Essential Medicines (EML) 2017 and matched the same molecule, dose, and formulation. A supplemental analysis was conducted to examine whether the active ingredient (not considering dose or formulation) was procured for certain disease categories.

**Price.** Price per unit was based on the price calculated by the procurers as published on the original procurement lists and converted from local currency to US dollars. Prices were then sorted within each list from highest to lowest.

## RESULTS

### Identification and consolidation of procurement data

Four procurement lists were obtained for this analysis from procurers A (year 2017), B (2015–2017), C (2015–2017), and D (2016), either as Excel documents or in portable file format (PDF). In all, 4 707 medicines with unique combinations of manufacturers, doses, and formulations were included in the database for the respective periods. The highest number of procured medicines was found for procurer A (2 607), followed by B (877), D (755), and C (468). There were similarities and differences in the information provided among the lists. Each list included the International Nonproprietary Name (INN), dose, formulation, and price of each procured medicine. Manufacturer and volume procured was included but this was inconsistent. Prices from procurer D were reported in the local currency, whereas procurer C reported prices in US dollars. The lists for procurers B and A also included the cost of insurance and freight. The product manufacturer was not provided for all products procured by B (and was thus excluded from all-manufacturer analyses), and volume procured was not included in procurer D’s list.

**FIGURE 1.**
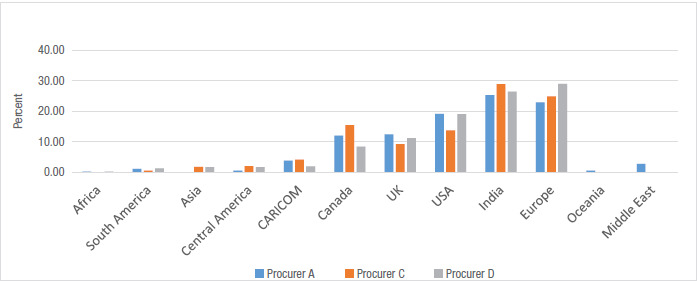
Percentage of medicines procured by manufacturer headquarters for CARICOM procurers

***Note:*** UK, United Kingdom: USA, United States of America.

***Source:*** Prepared by the authors based on the study data

**Location of manufacturer headquarters.** Medicines procured on each list were produced primarily by manufacturers with headquarters in countries with RRAs: Canada, Europe, United Kingdom, and United States of America. Products from these countries made up approximately two-thirds of procured medicines by procurers D (67%), A (66%), and C (63%). Manufacturers from India supplied more than a quarter of the procured medicines for procurers C (29%), D (26%), and A (25%). Small proportions of medicines were procured from manufacturers based in CARICOM for procurers C (4%), A (4%), and D (2%). Medicines from manufacturers based in South America and Central America constituted less than 2% of procured medicines, respectively (Figure 1).

**FIGURE 2.**
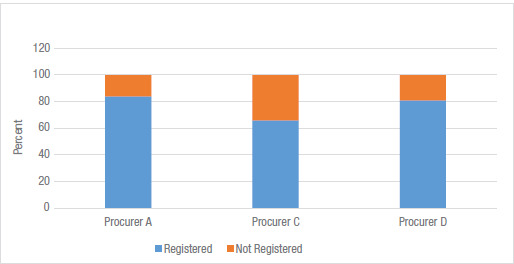
Percentage of medicines from manufacturers with at least one prescription medicine approved by a reference regulatory authority

***Source:*** Prepared by the authors based on the study data

**Regulatory history.** The proportion of manufacturers with at least one prescription medicine approved by a regulatory authority in Australia, Canada, United Kingdom, or United States of America ranged from 66% (C) to 84% (A) (Figure 2).

A sub-analysis of Indian manufacturers indicated only 39%–51% of these medicines were from companies with at least one prescription medicine approved by an RRA across procurers.

**FIGURE 3.**
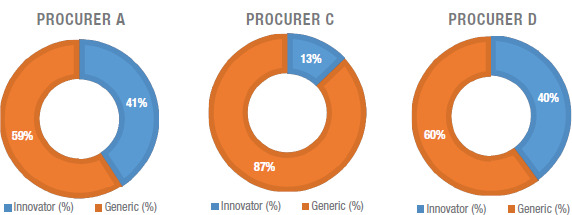
Percentage of medicines procured by manufacturer type

***Source:*** Prepared by the authors based on the study data

**FIGURE 4.**
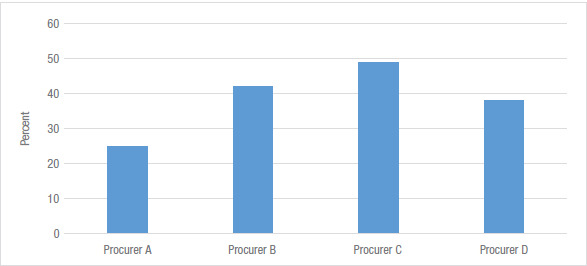
Percentage of procured medicines included in WHO 20th Essential Medicines List

***Source:*** Prepared by the authors based on the study data

**Company type.** Procurer C procured 87% of medicines from generic manufacturers, in comparison with procurers D (60%) and A (59%) (Figure 3).

**WHO EML.** Procurer C procured the highest proportion of essential medicines, followed by procurers B, D, and A (Figure 4).

A sub-analysis of the percentage of molecules procured compared with the EML molecules recommended by disease categories for NCDs, because these are a major challenge in the region, found there is a wide range of procurement. The EML recommends 14 medicines for mental and behavioral disorders, 22 for cardiovascular conditions, and 51 antineoplastics and immunosuppressants. There are similar levels of procurement for mental and behavioral disorders (range 64%–71%). There is a wider difference across cardiovascular medicines, from 55% to 82%. However, the widest difference is seen across the antineoplastics and immunosuppressants, where procurers C and B procure 25% and 45% of recommended molecules, versus 63% and 69% by procurers A and D, respectively.

**Price.** The five costliest medicines procured by procurers A, C, and D are shown in Table 1.

**TABLE 1.** Top-five costliest medicines by procurers A, C, and D

International nonproprietary name (INN)	Price/unit (US$)
Procurer A	
Octreotide acetate injection 20 mg	$1 948.98
Interferon beta solution for injection 80 mu/mL	$1 406.84
Tenecteplase solution for injection 50 mg	$1 372.52
Alteplase solution for injection 50 mg	$1 278.33
Interferon beta solution for injection 30 mcg/mL	$1 274.30
Procurer C	
Natamycin ophthalmic solution 5%	$126.72
Anti-D (RHO) immunoglobulin solution for injection 300 mcg	$113.10
Docetaxel solution for injection 80 mg/2 mL	$108.00
Sodium polystyrene sulfonate suspension 1.25 mg/5 mL	$65.00
Paclitaxel solution for injection 300 mg	$49.00
Procurer D	
Trastuzumab solution for injection 440 mg/20 mL	$1 792.04
Basiliximab powder for injection 20 mg	$1 715.75
Bevacizumab solution for injection 400 mg/16 mL	$1 635.00
Octreotide solution for injection 20 mg	$1 621.93
Rituximab solution for injection 500 mg/50 mL	$1 592.91

***Source:*** Prepared by the authors based on the study data

## DISCUSSION

### Common medicines sources

Companies with headquarters in North America and Europe manufacture the majority of medicines procured, ranging from 63% to 67% across procurers A, C, and D. This would appear to augur well for the quality of medicines, since many of these manufacturers produce medicines for highly regulated markets. However, where companies produce different tiers of products depending on the destination, even when they are the same active ingredient, dose, and formulation, this advantage is lost. This can include having a higher standard of production for strictly regulated markets, and a lower standard for poorly regulated markets, or for medicines that are for export only, which are often not made to the same standard as for domestic use (i.e., sometimes referred to as “for export only”) (15). CARICOM countries are particularly vulnerable because of their limited regulatory capacity and small market sizes, which are less commercially attractive than larger markets (16).

It is therefore critical for CARICOM countries to implement mechanisms that verify medicines are the same, to ensure quality medicines are procured. WHO states this occurs when a medicine is the same in respect to starting materials, manufacturing process, and manufacturing site of the product authorized by the RRA (17). Governments should compare the local submission to the RRA-approved product across critical categories including: qualitative and quantitative formulation; manufacturing sites for the Active Pharmaceutical Ingredient (API) and Finished Pharmaceutical Product (FPP); and product information, among others (18). Currently, no CARICOM countries conduct these verification checks.

### Regulatory history of manufacturers

The regulatory history of manufacturers is also important to consider. Companies with headquarters in India are the largest suppliers of medicines to the Caribbean outside of Canada, Europe, United Kingdom, and United States of America, making up a quarter of purchases. However, challenges with regulation of such products have been well documented (19, 20) and this could have implications for quality. Only 39%–51% of the products supplied come from companies that have at least one prescription product approved in an RRA, compared with 66%–84% of products supplied when all manufactures are considered.

Ideally, all medicines sold in a country should be reviewed by the national regulatory authority (NRA) for safety, quality, and efficacy before authorization for legal sale and procurement. However, due to the small nature of CARICOM states, regulatory capacity is limited, and some countries do not have NRAs. The national or regional procurer may be the only quality check on the product before provision to the public sector (21). Yet in many cases, Caribbean procurers authorize at the level of the manufacturer or importer, doing checks on business and logistical capacity, while scrutiny of quality at the individual product level varies. It may be helpful for procurers to check whether a company submitting products for procurement is known to have approvals from an SRA or RRA by looking at publicly available marketing authorization databases (with the caveats about versions mentioned above). If there are no approvals, it may indicate either an unwillingness or an inability to conform to international regulatory quality standards. Or it may be the case that a company is focused on a local or regional market. In places where regulation is limited, locally or regionally produced products should be approached carefully to understand the degree of regulatory oversight.

### Rational use

In terms of affordability and rational use, the procurers assessed in this paper purchase anywhere from 60% to 87% of products from companies that manufacture generic medicines. Procurer C procures almost all from companies that manufacture generic medicines (87%), while procurers D and A procure about 60%. For the latter two, this suggests there could be additional savings to pharmaceutical budgets if generic medicines are procured when quality versions are offered (22).

Affordability and rational use are also impacted by the degree of procurement of essential medicines. Procurement of WHO essential medicines does not exceed 50% of the lists of any of the procurers studied. Some procurers may purchase different doses or formulations than what is listed in the EML. Procurers also purchase different unique molecules than those listed in the EML, including within the same therapeutic category. Reducing the size of these formularies, while increasing the percentage of EML products, has been, and continues to be a worthwhile goal, but also a major challenge (23).

Another finding is that procurers may do better in procurement of some essential medicines than others. For example, procurer C purchases the highest percentage of essential medicines (50%) out of total medicines procured. However, it also procures a lower percentage of some categories of essential medicines, including about 25% of recommended antineoplastics and immunosuppressants. Procurer A procures the lowest percentage of EML-listed medicines out of total procurement at ~25%, but this is in part affected by its high overall number of medicines procured, at 2 607 versus 877, 468, and 755 for procurers D, C, and B, respectively.

These contrasting procurement situations may point to different economic environments. For example, procurer C appears to have a procurement policy that focuses on purchasing generic essential medicines, which lowers pharmaceutical costs. A limited budget may also explain in part why it does not purchase as many of the EML-recommended cancer medicines, such as those in the expensive biotherapeutic category. In contrast, procurers A and D serve high-income countries in the Caribbean as measured by gross domestic product (GDP) per capita, and therefore have more financial resources to purchase products. This is evident in the pricing analyses, which show a difference between the five highest priced medicines between procurers C, D, and A.

### Regional mechanisms

Recent policy initiatives may positively impact the pharmaceutical situation in CARICOM. Although voluntary, the CARPHA/CRS focuses on helping CARICOM states with marketing authorization and post-market surveillance. It requires products to be approved by an RRA, including WHO Prequalification, and verifies the medicines intended for the local market are the same in terms of manufacturing process, site, and starting materials. The CRS focuses its efforts on essential medicines, which are mostly generic. Thus, the CRS could be a mechanism to drive quality and rational use of medicines in CARICOM countries in the future. The initiative has recommended more than 100 products since April 2017, including medicines for HIV and noncommunicable diseases (24), but few national governments have procured them, signaling a future opportunity.

### Limitations

Limitations to these analyses include missing data from certain countries. Second, the large volume of data analyzed introduces the possibility of errors, including through artifacts in reporting on procurement lists. Third, much of the analysis revolves around percentage calculations of the denominators of summed medicines included on each list. If there are errors in terms of removal of non-medicines or repetitions of medicines, then the denominators will be affected. Fourth, the methodologies used to add information beyond what is included in the publicly available lists could introduce errors. For example, activities such as determining company headquarters may underestimate the true origin of products because it does not identify location of the manufacturing plant. Other limitations include the determination of generic versus innovator products, which does not comment on bioequivalence or patent status. Additionally, the currently available data on prices are not standardized across jurisdictions, making it difficult to compare; however, the top-five analyses conducted in this paper are internally valid because they can be sorted from high to low in each list. Finally, this analysis does not indicate how many of these medicines are actually available to patients.

### Conclusion

The procurement analyses in this paper identify vulnerabilities and opportunities in the procurement situation of CARICOM states, particularly related to quality and rational use of medicines. Governments or other partners can continue these analyses in the future, and they would be made easier if procurers could harmonize information to facilitate comparisons across countries.

## Disclaimer.

Authors hold sole responsibility for the views expressed in the manuscript, which may not necessarily reflect the opinion or policy of the *RPSP/PAJPH* and/or the Pan American Health Organization.

## Footnotes

* CARICOM Member States are: Antigua and Barbuda, Bahamas, Barbados, Belize, Dominica, Grenada, Guyana, Haiti, Jamaica, Montserrat, Saint Lucia, Saint Kitts and Nevis, Saint Vincent and the Grenadines, Suriname, and Trinidad and Tobago; Associate Member States are: Anguilla, Bermuda, British Virgin Islands, Cayman Islands, and Turks and Caicos Islands.

## References

[1] 1. World Health Organization. Access to medicines and vaccines report by the Director General. Geneva: WHO; 2019. Available from: https://apps.who.int/gb/ebwha/pdf_files/WHA72/A72_17-en.pdf [Accessed 2020 Nov 15].

[2] 2. Pan American Health Organization. Directing Council resolution on access and rational use of strategic and high-cost medicines and other health technologies. Washington DC: PAHO; 2016. Available from: https://www.paho.org/hq/dmdocuments/2016/CD55-10-e.pdf [Accessed 2020 Nov 15].

[3] 3. Pan American Health Organization; Caribbean Community and Common Market. Caribbean Pharmaceutical Policy. Washington DC: PAHO/CARICOM; 2013. Available from: https://carpha.org/Portals/0/Documents/Caribbean_Pharmaceutical_Policy-2013.pdf [Accessed 2020 Nov 15].

[4] 4. Caribbean Public Health Agency [Internet]. [Port of Spain]: CARPHA; c2020 [Accessed 2020 Nov 15]. The Caribbean Regulatory System. Available from: http://new.carpha.org/CRS/Caribbean-Regulatory-System

[5] 5. World Health Organization [Internet]. Geneva: WHO; [no date] [Accessed 2020 Nov 15]. Development of Country Profiles and monitoring of the pharmaceutical situation in countries. Available from: http://www.who.int/medicines/areas/coordination/coordination_assessment/en/

[6] 6. Preston C, Chahal HS, Porrás A, Cargill L, Hinds M, Olowokure B, et al. Regionalization as an approach to regulatory systems strengthening: a case study in CARICOM member states. Rev Panam Salud Publica. 2016;39(5):262–8. 27706404

[7] 7. Pan American Health Organization [Internet]. Washington DC: PAHO; 2019 Jul 9 [Accessed 2020 Nov 15]. PRAIS. Belize became the first country in the CARICOM block of countries to publish a list of registered medicines. Available from: https://prais.paho.org/en/belize-became-the-first-country-in-the-caricom-block-of-countries-to-publish-a-list-of-registered-medicines-2/

[8] 8. Organisation of Eastern Caribbean States [Internet]. Castries: OECS; c2016 [Accessed 2020 Nov 15]. Pharmaceutical Procurement Service. Available from: https://www.oecs.org/our-work/knowledge/library/pps

[9] 9. World Health Organization. Model List of Essential Medicines. Geneva: WHO; 2017. Available from: https://www.who.int/medicines/publications/essentialmedicines/en/ [Accessed 2020 Nov 15].

[10] 10. Jamaica, National Health Fund [Internet]. Kingston: NHF; c2020 [Accessed 2020 Nov 15]. Schedule of pharmaceutical awards 2015/2017. Available from: https://www.nhf.org.jm/

[11] 11. Trinidad and Tobago, National Insurance Property Development Company Limited [Internet]. Port of Spain: NIPDEC; c2020 [Accessed 2020 Nov 15]. Price list booklet 2016. Available from: https://nipdec.com/our-services/

[12] 12. Organisation of Eastern Caribbean States [Internet]. Castries: OECS; c2016 [Accessed 2020 Nov 15]. Pharmaceutical Procurement Service. List of primary awards 2015-2017. Available from: https://www.oecs.org/our-work/knowledge/library/pps

[13] 13. World Health Organization. Marketing Authorization of Pharmaceutical Products with Special Reference to Multisource Generic Products. Geneva: WHO; 2011. Available from: https://apps.who.int/iris/bitstream/handle/10665/44576/9789241501453_eng.pdf [Accessed 27 November 2020].

[14] 14. World Health Organization. Proposal for updating the definition of Stringent Regulatory Authority. Geneva: WHO; 2017. Available from: http://www.who.int/medicines/areas/quality_safety/quality_assurance/SRA_QAS17-728Rev1_31082017.pdf [Accessed 2020 Nov 15].

[15] 15. Caudron J-M, Ford N, Henkens M, Macé C, Kiddle-Monroe R, Pinel J. Substandard medicines in resource-poor settings: a problem that can no longer be ignored. Trop Med Int Health. 2008;13(8):1062–72. 10.1111/j.1365-3156.2008.02106.x 18631318

[16] 16. Pan American Health Organization. Regulatory system models for small states/markets with limited resources: concept note and recommendations. Washington DC: PAHO; 2020. Available from: https://iris.paho.org/bitstream/handle/10665.2/52387/pahohssmt200003_eng.pdf [Accessed 2020 Nov 15].

[17] 17. World Health Organization. Good reliance practices in regulatory decision-making: high-level principles and recommendations. Geneva: WHO; 2020. Available from: https://www.who.int/medicines/areas/quality_safety/quality_assurance/QAS20_851_good_reliance_practices.pdf [Accessed 2020 Nov 15].

[18] 18. World Health Organization. Expert Committee on Specifications for Pharmaceutical Preparations, fifty-second report. Geneva: WHO; 2018. Available from: https://apps.who.int/iris/bitstream/handle/10665/272452/9789241210195-eng.pdf [Accessed 2020 Nov 15].

[19] 19. Altstedter A, Edney A. Bloomberg [Internet]. [New York]: Bloomberg L.P.; 2019 Jan 31 [Accessed 2020 Nov 15]. Culture of ‘Bending Rules’ in India Challenges U.S. Drug Agency. Available from: https://www.bloomberg.com/news/features/2019-01-31/culture-of-bending-rules-in-india-challenges-u-s-drug-agency

[20] 20. National Academies of Sciences, Engineering, and Medicine. Regulating medicines in a globalized world: the need for increased reliance among regulators. Washington DC: The National Academies Press; 2020. Available from: https://www.nap.edu/catalog/25594/regulating-medicines-in-a-globalized-world-the-need-for-increased [Accessed 2020 Nov 15]. 32293828

[21] 21. Preston C, Freitas Dias M, Peña J, Pombo ML, Porrás A. Addressing the challenges of regulatory systems strengthening in small states. BMJ Glob Health. 2020;5(2):e001912. 10.1136/bmjgh-2019-001912 PMC7053784 32180997

[22] 22. Cameron A, Laing R; World Health Organization. Cost savings of switching private sector consumption from originator brand medicines to generic equivalents. World Health Report (2010) Background Paper No 35. Geneva: WHO; 2010. Available from: https://www.who.int/healthsystems/topics/financing/healthreport/35MedicineCostSavings.pdf [Accessed 2020 Nov 15].

[23] 23. Management Sciences for Health. Managing medicine selection. In: MDS-3: Managing Access to Medicines and Health. Arlington VA: MSH; 2012. Available from: https://www.msh.org/sites/msh.org/files/mds3-ch16-selection-mar2012.pdf [Accessed 2020 Nov 15].

[24] 24. Caribbean Public Health Agency [Internet]. [Port of Spain]: CARPHA; c2020 [Accessed 2020 Nov 15]. CARICOM MEMBER STATE ENGAGEMENT. Recommended medicines list. Available from: http://new.carpha.org/CRS/CARICOM-Member-State-Engagement

